# Gut microbes in cerebrovascular diseases: Gut flora imbalance, potential impact mechanisms and promising treatment strategies

**DOI:** 10.3389/fimmu.2022.975921

**Published:** 2022-10-31

**Authors:** Xuelun Zou, Leiyun Wang, Linxiao Xiao, Sai Wang, Le Zhang

**Affiliations:** ^1^ Department of Neurology, Xiangya Hospital, Central South University, Changsha, Hunan, China; ^2^ Department of Pharmacy, Wuhan First Hospital, Wuhan, China; ^3^ Department of Spine Surgery, Xiangya Hospital, Central South University, Changsha, Hunan, China; ^4^ National Clinical Research Center for Geriatric Disorders, Xiangya Hospital, Central South University, Changsha, Hunan, China; ^5^ Multi-Modal Monitoring Technology for Severe Cerebrovascular Disease of Human Engineering Research Center, Changsha, Hunan, China

**Keywords:** cerebrovascular diseases, ischemic stroke, intestinal microbiome, fecal bacteria transplantation, inflammation, immunology

## Abstract

The high morbidity, mortality, and disability rates associated with cerebrovascular disease (CeVD) pose a severe danger to human health. Gut bacteria significantly affect the onset, progression, and prognosis of CeVD. Gut microbes play a critical role in gut-brain interactions, and the gut-brain axis is essential for communication in CeVD. The reflection of changes in the gut and brain caused by gut bacteria makes it possible to investigate early warning biomarkers and potential treatment targets. We primarily discussed the following three levels of brain-gut interactions in a systematic review of the connections between gut microbiota and several cerebrovascular conditions, including ischemic stroke, intracerebral hemorrhage, intracranial aneurysm, cerebral small vessel disease, and cerebral cavernous hemangioma. First, we studied the gut microbes in conjunction with CeVD and examined alterations in the core microbiota. This enabled us to identify the focus of gut microbes and determine the focus for CeVD prevention and treatment. Second, we discussed the pathological mechanisms underlying the involvement of gut microbes in CeVD occurrence and development, including immune-mediated inflammatory responses, variations in intestinal barrier function, and reciprocal effects of microbial metabolites. Finally, based on the aforementioned proven mechanisms, we assessed the effectiveness and potential applications of the current therapies, such as dietary intervention, fecal bacterial transplantation, traditional Chinese medicine, and antibiotic therapy.

## Introduction

Given their high mortality and disability rates, cerebrovascular diseases (CeVDs) seriously threaten human health and place significant strain on healthcare systems, particularly in developing nations (1, 2). CeVDs include ischemic stroke (IS), intracerebral hemorrhage (ICH), cerebral cavernous malformation (CCM), intracranial aneurysm (IA), and cerebral small vessel disease (CSVD), among others. IS, which accounts for approximately 80% of CeVD occurrences, has the highest incidence and prevalence (3). Additionally, due to its high mortality, impairment rates (between 30% and 40%), and prevalence (between 10% and 15%), ICH is the second most common CeVD after IS (3). CCM and IA are significant risk factors for ICH (4, 5). They may bleed and rupture unexpectedly, causing critical neurological illnesses and a significant disease burden (4, 5).

Patients’ quality of life is significantly reduced by CSVD, which makes them prone to dementia, stroke, and cognitive dysfunction (6). In addition, pathophysiological changes, such as transient and permanent cessation of oxygen supply to brain tissues and the energy metabolism of brain cells, are the results of an IS. Furthermore, an IS leads to excitotoxicity, oxidative stress, neuroinflammation, apoptosis, amyloid production, tau protein dysfunction, and other diseases (7–10). In turn, these diseases cause neurological deficits, changes in memory and intelligence, movement disorders, tissue compression, and other systemic and local brain function damage (7–10). In addition, increased intracranial pressure, hematoma compression, and severe brain edema are all caused by ICH, which leads to neuroinflammation, apoptosis, membrane depolarization, mitochondrial dysfunction, and other negative effects (11–16). Finally, patients may have a poor prognosis with impaired motor function, cognitive impairment, seizure-like epilepsy, and impaired consciousness (11–16). Therefore, early diagnosis and treatment of CeVD have important clinical implications.

Diet has been identified as a major risk factor for CeVD, and improper diet and nutrition are strongly associated with the likelihood of a first stroke (17, 18). Rapid economic development has progressively altered people’s diets, and the consumption of foods high in cholesterol, such as red meat and egg yolks, has increased dramatically, resulting in a 26.6% increase in the stroke mortality rate (18, 19). Research on how diet affects CeVD has shown that gut microorganisms are primary regulators (18, 20). Intestinal microbes refer to the many microorganisms residing in the human gastrointestinal tract that rely on the human intestinal environment for survival and aid in the execution of immunological, metabolic, and endocrine activities (21).

**Figure 1:**
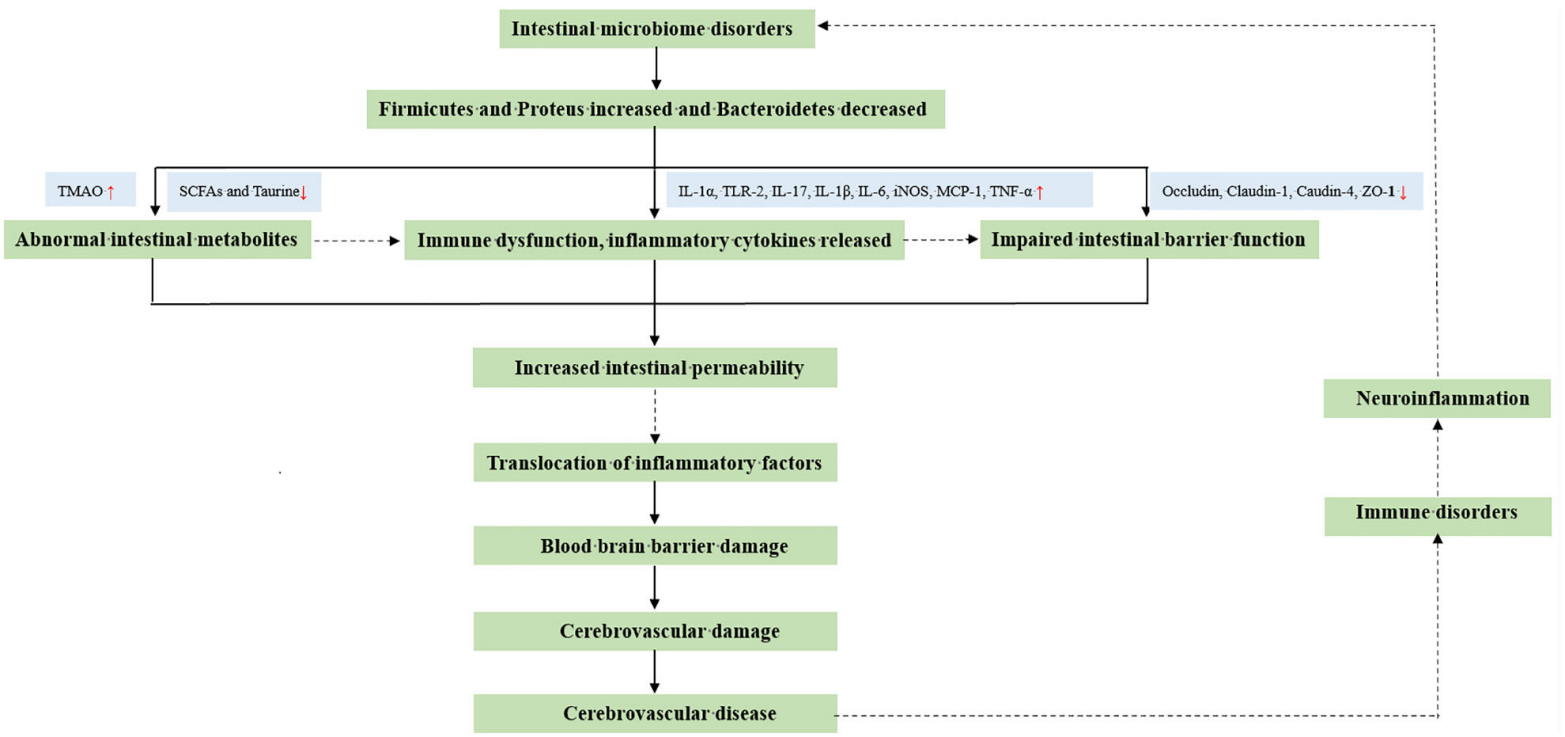
Potential impact mechanism between intestinal microorganisms and cerebrovascular diseases.

Therefore, we extensively surveyed the literature on gut microbes and CeVD from 2012 to date (**Figure 1**). First, we summarized and analyzed the core microbes affecting CeVD among the various gut microbes and the microbial differences affecting various CeVDs. Second, we explored the interaction mechanisms between gut microbes and CeVD, including immunomodulation of the inflammatory response, gut barrier failure, and bidirectional effects of microbial metabolites. Finally, we explored the efficacy and applicability of existing CeVD therapies at the gut microbial level based on the pathways mentioned above. These therapies include flora transplantation, traditional Chinese medicine (TCM), and antibiotic therapy. Research on gut microbes and even the gut-brain axis ultimately aims to solve the problems associated with CeVD and minimize the threat of these diseases.

## Imbalance of gut microbes

The evolution of the intestinal microbiota began with the colonization of parthenogenic anaerobic bacteria (*Denatobacteria*), followed by the growth of anaerobic bacteria (*Lactobacillus* and *Bifidobacteria*), and then evolved into diverse bacteria and various species of *Bacteroidetes* survived together (22, 23). With solid food consumption, intestinal microbes form a stable colony, which is then classified into three types according to their function. “Beneficial bacteria” help the body absorb nutrients, boost vitamin synthesis, resist the invasion of pathogenic microbes and toxic metabolites, and preserve the host’s homeostasis (22, 23). However, variations in beneficial bacteria can result in malnutrition, trace element insufficiency, systemic immunological problems, and pathological damage. The second category consists of “neutral bacteria,” which reside in the gut but are nonfunctional and have minimal health effects (22, 23). The third category is “dangerous bacteria,” which frequently impairs intestinal function, creates toxic compounds in the host, and weakens the body’s immune system (22, 23). Typically, these three types of bacteria coexist without causing any harm to their host’s health. The three groups of intestinal microorganisms are not mutually exclusive, though. The “beneficial bacteria,” “ neutral bacteria,” and “ dangerous bacteria” may transformation between states of the intestinal microbes especially in different diseases.

The dynamic equilibrium of the intestinal flora can be disrupted by immune system dysfunction or fluctuations in the intestinal environment. The amount of “beneficial bacteria” will decrease or exhibit abnormal functions, while the “dangerous microorganisms” reproduce rapidly (24–27). The human body absorbs numerous disease-causing chemicals through the digestive tract (24–27); this affects digestive function and the gut microbial balance. With intestinal ecological imbalance, intestinal mucosal immune function is disrupted, gastrointestinal permeability increases, massive bacterial translocation occurs, inflammatory factors spread, and intestinal immune dysfunction and pathogen resistance are diminished (24–27). Furthermore, changes in the dominant flora of gut microbes can also result in changes in gastrointestinal metabolites, such as a decrease in short-chain fatty acids (SCFAs) and an increase in trimethylamine-N-oxide (TMAO) (27–29). These metabolites are closely associated with neuroinflammation, post-stroke infection, and secondary brain injury. When intestinal microbial homeostasis is dysregulated, intestinal inflammatory factors such as T helper type (Th) 1, Th17, and interleukin IL-6 are released in large amounts. The release of these inflammatory factors results in intestinal permeability changes, barrier dysfunction, extravasation of inflammatory substances into peripheral blood, and transportation to the blood-brain barrier (BBB). Ultimately, they act on the cerebrovascular system and play a crucial role in CeVD occurrence, development, and prognosis (30–33). In CeVD research (34–37), the clinical efficacy of fecal bacterial transplantation (FMT), traditional Chinese medicine intervention, antibiotic intervention, and SCFA supplementation remains contentious.

## Abnormal abundance of the intestinal microbiome in CeVD

### Intestinal flora imbalance in IS

**Figure 2:**
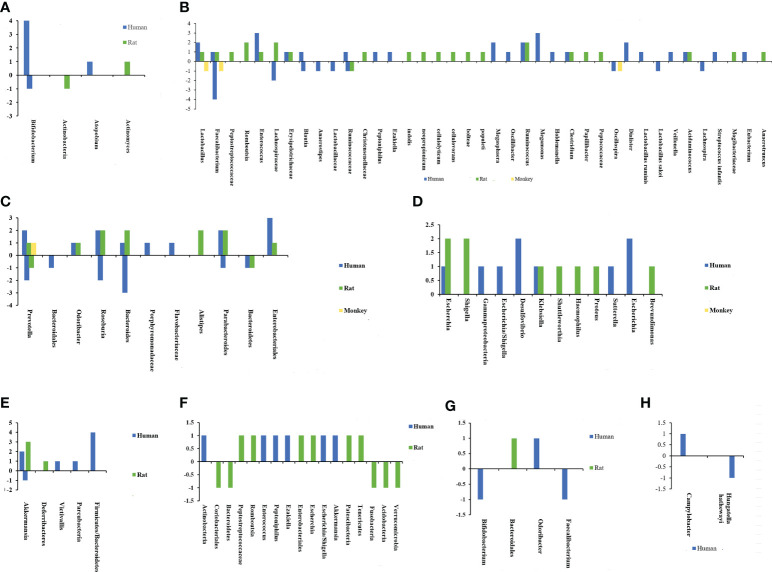
Chart of intestinal flora changes in cerebrovascular diseases. The abscissa represents different bacteria, and the ordinate shows the number of changes in flora. **(A)** Changes of actinobacteria after ischemic stroke. **(B)** Changes of Firmicutes after ischemic stroke. **(C)** Changes of Bacteroidetes after ischemic stroke. **(D)** Changes of Proteobacteria after ischemic stroke. **(E)** Changes of other bacterias after ischemic stroke. **(F)** Changes of intestinal flora after intracerebral hemorrhage. **(G)** Changes of intestinal flora after cerebral cavernous hemangioma. **(H)** Changes of intestinal flora after intracranial aneurysm.

**Figure 3:**
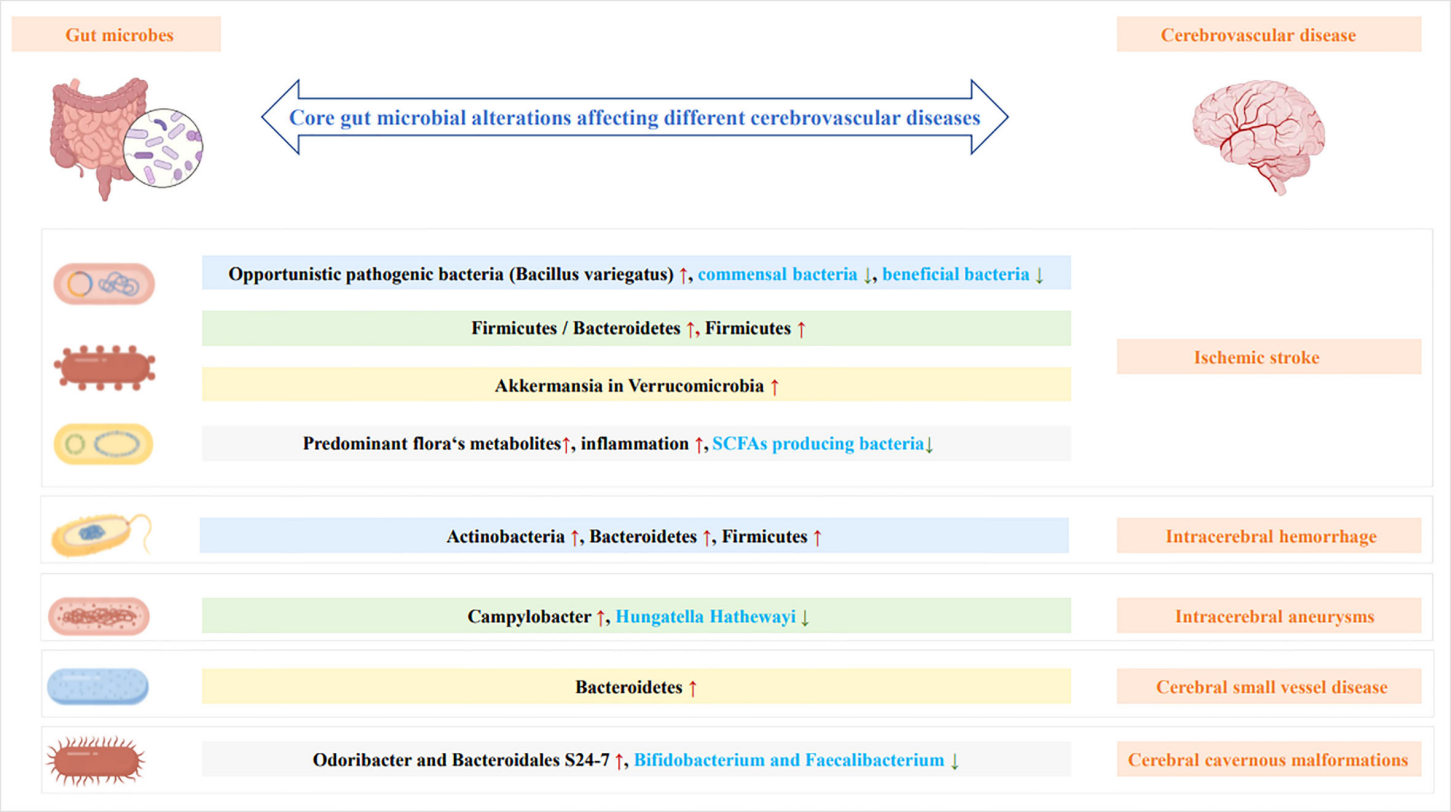
The change in core gut microbes influences different cerebrovascular diseases.

Based on the above background, the specific composition of microbiota associated with susceptibility to CeVD is beneficial in understanding the disease pathogenesis. In a series of case-control and animal model studies (20, 24, 25, 27, 29, 38–61) a correlation between IS and altered intestinal microflora was found. **Figures 2**, **3** and **Supplementary Table 1** show that the core florae of IS lesions are *Actinobacteria*, *Lentisphaerae*, *Thickobacteria*, and *Bacteroides* (20, 24, 25, 27, 29, 38–61). *Proteobacteria* and *Firmicutes* showed an overall increasing trend, while *Bacteroidetes* showed a decreasing trend, and there were some contradictions in the changes in *Actinobacteria* (20, 24, 25, 27, 29, 38–61). In addition, there were changes in *Verrucomicrobia*, *Lentisphaerae*, *Candidatus*, *Parcubacteria*, *Deferribacteres*, and other species of bacteria (20, 27, 41–44, 48, 54). However, there were some differences in the changes in different bacteria, different species of bacteria, and different lesion periods within each group.

#### Relationship between IS prevention and treatment and the enrichment of opportunistic pathogens in gut bacteria

One study has reported that before a stroke occurs, the abundance of *Enterobacteriaceae* and *Veillonellaceae* species increased in the high-risk IS group, indicating that an increase in the abundance of opportunistic infections is related to an increased risk of IS (38). Using 16S rRNA V4 tags, Illumina sequencing revealed a significant difference between IS and asymptomatic patients. In IS, the incidence of opportunistic gut pathogens, such as *Enterobacter*, *Megasphaera*, *Oscillibacter*, and *Desulfovibrio*, increased. Simultaneously, symbiotic and beneficial bacteria such as *Bacteroides*, *Prevotella*, and *Faecalibacterium* were diminished (27, 62). This alteration is also confirmed in atherosclerotic stroke, characterized by overgrowth of gut pathogens (e.g., *Desulfovibrio* and *Enterococcus*), opportunistic pathogens (e.g., *Eubacterium*), and a few commensals or beneficial species (e.g., *Bifidobacterium* and *Lactobacillus*) (39). In addition, the traditional Chinese medicine Tanhuo decoction treatment for IS focuses on (i) promoting the growth of symbiotic flora and beneficial bacteria, (ii) competitively inhibiting the growth of opportunistic pathogenic bacteria, (iii) reducing aseptic inflammation and platelet aggregation, and (iv) enhancing the host’s gut metabolism and body immunity (63). In addition to directly impacting stroke, the accumulation of opportunistic infections is associated with the incidence and severity of post-stroke pneumonia (64). After gut microbiota dysbiosis, opportunistic pathogenic bacteria flourish, particularly *Proteobacteria deformation* (primarily *Enterobacteriaceae*), whose excessive proliferation is a sign of epithelial dysfunction and intestinal microbial structural instability, which can also exacerbate inflammation and invasion of exogenous pathogens (65, 66). Environmental or host factors (acute or chronic inflammation) play a selective role in altering homeostasis and causing dysbiosis in gut microbes (66). It is often accompanied by an outbreak of *Proteobacteria deformation* in the gut (66). The inability of the host to sustain commensal *Proteobacteria deformation* in low quantities or the weakened resistance of the colonizing microbiome can result in uncontrolled growth of *Proteobacteria deformation*, which can exacerbate the damage caused by inflammation and invasion by foreign pathogens (66). Consequently, cutting the malignant feedback loop could be a viable intervention method for preventing IS (66).

#### Association of IS lesions with dysregulation of *Firmicutes*/*Bacteroidetes* ratio

*Firmicutes* and *Bacteroidetes* are two of the main gut microbial species. Both are obligate anaerobic bacteria that can adapt to the anaerobic environment of the intestines and ferment compounds, such as butyrate, which impact the human body (40, 41, 67, 68). The *Firmicutes*/*Bacteroidetes* ratio was originally discovered in obese people, demonstrating an increase in *Firmicutes* and a decrease in *Bacteroidetes* (69, 70). Furthermore, there was a substantial correlation between the ratio of *Firmicutes* to *Bacteroidetes* and the BMI of adults (69, 70). Changes in the ratio of *Firmicutes* to *Bacteroidetes* have also been reported in IS (40). The *Firmicutes*/*Bacteroidetes* ratio increased significantly in elderly mice with IS with intestinal ecological imbalance. Force-feeding young mice with an increased *Firmicutes*/*Bacteroidetes* ratio increased their mortality after IS. In addition, reducing the intestinal *Firmicutes*/*Bacteroidetes* ratio of elderly mice increased their survival rate and facilitated functional recovery (40). An increased *Firmicutes*/*Bacteroidetes* ratio was also observed in the intestinal flora of mice with IS, including reproductively aged animals with IS (41, 67, 68). **Figures 2A–E**) depicts the microbiological alterations in IS. *Firmicutes* exhibited a generally increasing trend, and the ratio of *Firmicutes* to *Bacteroidetes* increased (40, 41, 67, 68). A reduced *Firmicutes*/*Bacteroidetes* ratio is connected with a slim phenotype, youth, cardiovascular health, and a well-balanced immune system, all of which are advantageous for the human body (71–73). The increase in *Firmicutes*/*Bacteroidetes* flora decreases the population of SCFA-producing bacteria and increases the population of lactic acid-producing bacteria (74–76). SCFAs are the energy source for approximately 60–70% of the colon epithelium, which is strongly associated with intestinal immune function and intestinal barrier function, which may be a key reason why the imbalance of *Firmicutes*/*Bacteroidetes* flora impacts IS (74–76).

#### Connection of IS prognosis with significantly elevated levels of *Akkermansia* and *Verrucomicrobia* observed in IS

*Akkermansia* colonizes the intestinal mucosa and regulates basal metabolism by generating mucin-degrading enzymes and utilizing mucin as a source of nitrogen and carbon in the epithelial mucous layer (42). *Akkermansia* and *Lactobacillus* growth is frequently observed in intestinal microbiota abnormalities following an IS (40–44). Both are beneficial bacteria that can increase the growth of other beneficial bacteria and create beneficial neuroactive chemicals, which may be related to a compensatory response secondary to the massive reduction of SCFA-producing bacteria, especially butyrate-producing bacteria (41, 77). *Akkermansia* may use mucin to produce high quantities of acetate, which the *Ruminococcus* family that produces butyrate can stimulate butyrate production (38, 42, 43). Butyrate can improve wound healing, reinforce intestinal epithelial cell integrity in mice with IS, and inhibit bacterial migration (38, 42, 43). In addition, butyrate can stimulate mucus production and *Reg3γ* expression in the colon, leading to microbiome reorganization (43, 78). The blood inflammatory markers IL-2, interferon-gamma (IFN-γ), IL-12P40, and monocyte chemoattractant protein-1 (MCP-1) can be reduced by artificially increasing the abundance of *Akkermansia* bacteria in the colon (79). In addition, it can improve intestinal tight junction proteins (claudin 3, block occluder, and cannabinoid receptor 1), reverse monooxygenase 3 containing linolenin in the liver, and promote the conversion of trimethylamine (TMA) to TMAO (79). These effects contribute to the stable state of the basal metabolism of the host and stimulate the generation of additional beneficial bacteria *via* diverse signaling pathways (79).

#### Connection of IS occurrence and severity to the predominant flora’s metabolites

IS is associated with bacteria with poor SCFA production (41). In contrast, another study found that SCFA-producing bacteria (*Odoribacter*, *Akkermansia*, *Ruminococcaceae* UCG-005, and *Victivallis*) were abundant in late IS. *ChristensenellaceAE* R-7 group, norank f Ruminococcaceae, and *Enterobacter* levels are associated with stroke severity (43). The amount of *ChristensenellaceAE* R-7 is positively associated with the outcome of patients with IS (43). Changes in the bacterial abundance of SCFAs may be associated with the severity and duration of stroke lesions. Furthermore, the relative abundance of SCFA-producing bacteria decreases in patients with stroke-induced cognitive impairment and depression (40, 80). SCFA supplementation can improve post-stroke recovery, cortical reconfiguration, and synaptic plasticity (81). Butyrate is an essential SCFA; its bacterial abundance corresponds to stroke severity (33, 43, 45). *Roseburia* was considerably enriched in butyrate in mild stroke patients (NIHSS score <4); conversely, *Erysipelotrichaceae incertae sedis* was abundant in non-mild stroke patients (43, 45). In addition, isobutyric acid, butyric acid, and 2-methyl butyric acid were negatively associated with the percentage change in NIHSS scores. Furthermore, isobutyric acid and 2-methyl butyric acid were favorably linked with NIHSS discharge (33). The abundance of butyrate-producing bacteria was also associated with post-stroke infection (29). Butyrate-producing bacteria were also associated with fasting blood glucose, and severe stroke was positively associated with stress hyperglycemia (fasting blood glucose/glycosylated hemoglobin ratio) (45). It has also been demonstrated that artificially increasing butyrate (sodium butyrate) reduces BBB damage and infarct size in diabetic mice with IS (40, 46).

### Intestinal flora imbalance in ICH, CSVD, IA, and CCM

#### Current lack of research on ICH, CSVD, IA, and CCM

Studies have reported that *Escherichia*/*Shigella*, *Peptoniphilus*, *Ezakiella*, and *Enterococcus* are more abundant in the bacterial flora after IS and ICH (29). ICH is primarily characterized by increased *Actinomycetes*, *Bacteroidetes*, and *Firmicutes* and minor alterations in other bacteria (25, 40, 48). Moreover, gut microorganisms change over time. After ICH, *Proteobacteria deformation* (*Escherichia coli*) grew significantly on the first day, whereas *Firmicutes* (*Romboutsia* and *Peptostreptococcaceae*) increased significantly on the third day and then continued to be dominated by an increase in *Firmicutes* (25). Intracerebral aneurysms are the primary cause of CeVD, including subarachnoid and intracranial hemorrhage (ICH). The decrease in *Hungatella hathewayi* (35) and the increase in *Campylobacter* (82) are the predominant microorganism changes in the intestines that influence the onset, progression, and prognosis of IA. These bacteria have not been found in other CeVD for the time being (35, 82). An increase in *Bacteroidetes* may play a role in the incidence of dementia after CSVD (83). In CCM, *Actinomycetes* (*Bifidobacterium*) and *Firmicutes* (*Faecalibacterium*) declined, whereas *Bacteroides* (*Odoribacter* and *Bacteroidales* S24-7) increased in the gut microbiome (49, 84).

## Gut-brain axis potential mechanism

**Figure 4:**
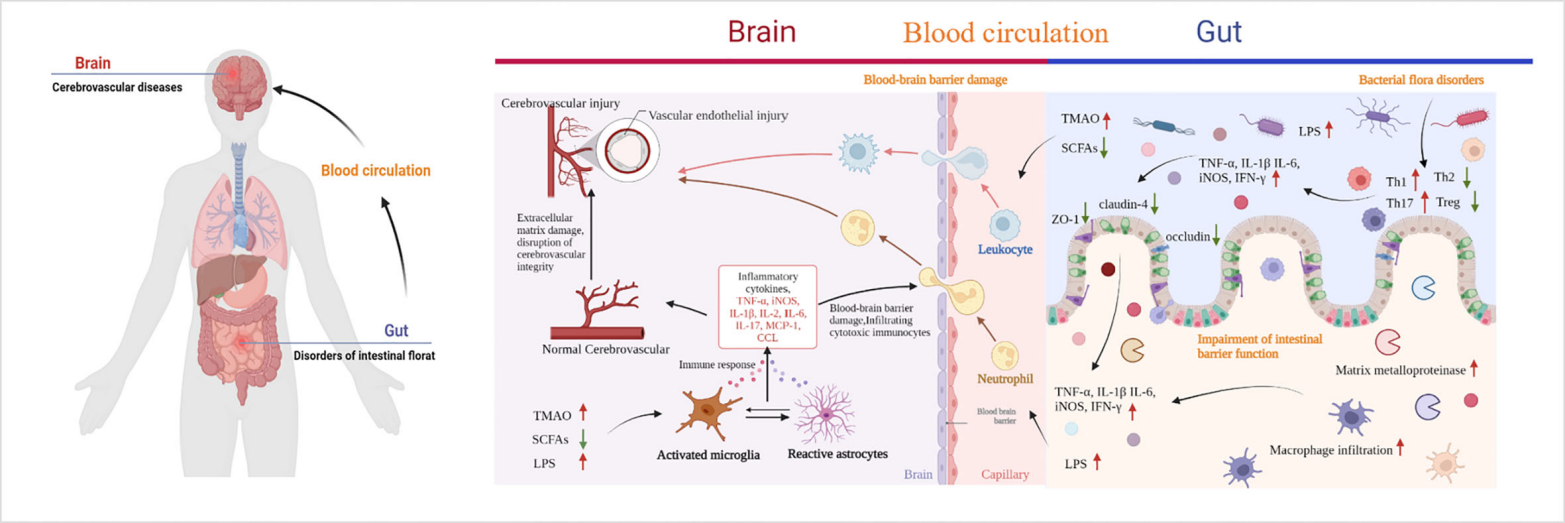
Pathways of cerebrovascular injury due to intestinal microbial imbalance. TMAO, trimethylamine-N-oxide; SCFAs, short-chain fatty acids; LPS, lipopolysaccharide; TLR4, toll-like receptor 4; CCL, chemokines. By Figdraw and BioRender.com.

The gastrointestinal tract is an important immune organ with the largest immune cell repertoire. This collection includes almost 70% of the immune system of the body. As mentioned before, CeVD is characterized by alterations in the different flora of intestinal microorganisms. Inflammatory responses and immune modulations follow various degrees of damage to the gut and brain. They lead to alterations in the intestinal barrier’s function and the impact of microbial metabolites on the brain through this barrier, which may be a way to combat CeVD in the future. Therefore, understanding the mechanisms underlying the lesions is the most effective way to identify targets for intervention (**Figure 4**).

### Inflammatory response and immune regulation in CeVD

During IS, the gut microbiota varies rapidly (minutes to hours). Multiple signals of intestinal homeostasis disorder, vagal nerve activation, and cerebral inflammatory signals stimulate intestinal myeloid cells to activate receptor 1. This worsens damage to the intestinal mucosal layer, modifies intestinal permeability, and permits intestinal cells to enter the bloodstream from the intestines (85, 86). When LPS and bacteria are translocated to the peripheral area, normal immune homeostatic processes in the gut can be damaged by the extravasation of immune cells into the brain tissue, which accelerates brain damage after stroke (87). After IS, bone marrow cells from peripheral triggering receptor expressed on myeloid cells-1(TREM1) induce a fast innate immune response that inhibits brain recovery processes, including glutathione metabolism, anti-inflammatory TREM2 signaling, and lysosomal degradation (86). LPS produced by Gram-negative bacteria translocate from the intestine to the systemic circulation after intestinal imbalances and increased intestinal permeability. Moreover, after IS, the BBB is damaged, allowing LPS to enter the brain and bind to inflammatory cytokines and TLR4 receptors on microglia (84, 87–90). Consequently, microglia and macrophages are hyperactivated, resulting in (i) exacerbated neuroinflammation and large amounts of inflammatory factors such as TNF-α, IL-1β, IL-6, TNF, and iNOS release, (ii) increased production of chemokines in the brain parenchyma, and (iii) subsequent infiltration of cytotoxic immune cells, including neutrophils, inducing neuronal apoptosis and brain injury (84, 87–90). The gut microbes can also be affected. There is a negative correlation between *Bifidobacterium* and IL-1, IL-2, IL-6, and hs-CRP levels (47). The elevated expression of the inflammatory cytokines IL-2 and IL-6 in the intestine and brain tissues of patients with post-stroke depression decreased following exercise intervention, which may be related to the enhanced expression of *Lactobacillus* and *Bifidobacteria* (91). Besides, the ratio of *Firmicutes* to *Bacteroidetes* was higher in older mice than in younger mice, and systemic proinflammatory cytokine levels were elevated (40). When *Bifidobacterium longum*, commensal *Clostridia*, *Eubacterium faecalis*, and *Lactobacillus fermentum* were transplanted into old mice, neuroinflammation and neurological impairments following stroke were mitigated to varying degrees (37). Except the direct effect, metabolites of dysbiosis, also promote the release of inflammatory factors, and the indirect effect of IS cannot be ignored. SCFAs have been associated with elevated levels of systemic proinflammatory markers IL-6, TNF-α, vascular cell adhesion molecule-1(VCAM-1), IL-17, and MCP-1 after stroke, which was associated with a high disease burden at discharge and the duration of inpatient recovery (33). Moreover, butyrate-producing bacterial abundance is an independent predictor of post-stroke infection (29).

IA rupture is associated with gut microbiota affecting the influx of macrophages within the aneurysm, matrix metalloproteinase (MMP) production, and the imbalance between pro-inflammatory and anti-inflammatory cells M1 and M2 (92, 93). After antibiotic inhibition of intestinal microorganisms, inflammation in cerebral arteries during aneurysm formation is suppressed, the number of macrophages is reduced, mRNA expression of inflammatory factors IL-1β, IL-6 and iNOS is decreased, and MCP-1 is reduced (93). In addition, the expression of the pro-inflammatory cytokines IL-6 and TNF-α was increased in animals transplanted with feces from IA patients (35). The increased gene expression of intestinal proinflammatory proteins IL-1, (toll-like receptor) TLR-2, and IL-17 in CSVD-affected animals facilitated the buildup of bacterial toxins and bacteria, triggered the progressive progression of gut inflammation into systemic inflammation, and consequently exacerbated BBB damage (30). However, in CCM, the cell wall component of gram-negative bacterial lipopolysaccharides, in conjunction with the intracellular adaptor protein complex defect resulting from the loss of function mutations of Krev interaction trapped protein 1, CCM2, and programmed cell death 10 genes, activates TLR4 in brain endothelial cells through *TLR4-Mekk3-KLF1/4* signal transduction (84, 88). CCM production is accelerated by activating innate immunity and co-mediating inflammation reactions with lipopolysaccharide (LPS) (84, 88). ICH significantly increases the intestinal inflammatory response by increasing the release of the inflammatory cytokines IL-1β, IL-6, and TNF-α, activating intracellular adhesion molecule-1 (ICAM-1) and other chemokines, including MCP-1 and CCL-5, MPO activity, and IL-1β concentration in the peripheral circulation, initiating the infiltration of inflamed cells into the intestine, leading to delayed small bowel motility and bowel obstruction (94–97). One study reports that intestinal inflammatory factors and T lymphocytes gradually infiltrate the ICH hematoma, and the number of CD4 T cells, CD8 T cells, and regulatory T lymphocytes (Tregs) increases (98, 99). Additionally, IFN-γ and IL-17 proinflammatory cytokine expression increases (99). Fourteen days after ICH, the mRNA levels of inducible nitric oxide synthase, tumor necrosis factor, and other proinflammatory indicators were significantly elevated (99).

### Intestinal barrier dysfunction

After separating the external intra-abdominal environment from the inside milieu of the gastrointestinal tract, the gastrointestinal barrier measures approximately 400 m^2^. This barrier serves a dual purpose: it permits the absorption of nutrients, water, and electrolytes and limits the host’s exposure to harmful lumen antigens (100). The mucosal, epithelial, and immunological layers constitute the intestinal barrier. CeVD in the three-layer barrier of the gut appeared to be damaged to varying degrees. It is also possible to track barrier disturbances during the early stages of CeVD in an is also possible to track barrier disturbances. The intestinal mucosa in the MCAO group showed cell edema, thickness, and shortening of the villi 3 hours postoperatively, which increased over time. In the early stages of IS, necrosis develops at the top of the villi and damages the intestinal mucosa within 24 hours (44, 50, 101). This results in altered gastrointestinal dynamics owing to villous shrinkage, crypt distortion, mucosal edema, and significant inflammatory cell infiltration.

Similarly, gastrointestinal motility is reduced to varying degrees (44, 50, 101). At the histomorphological level, substantial intestinal epithelial cell destruction, thickening and shortening of villi, the fusion of adjacent villi, and exposure of the lamina propria were noted following ICH (102). At the ultrastructural level, intestinal secretions increase, and epithelial cells are unevenly arranged, sparse, and fragmented at the surface of the villi (102). In addition, the intestinal mucus barrier was compromised in mice with CCM, and E-cadherin (CDH1) was reduced. However, the continuity of the epithelial cell adhesion molecule connection increased, and neither f-actin nor zonula occludens-1 (ZO-1) changed (103).

The morphological changes were external manifestations, while the internal disruption of barrier function was associated with (i) the lipid polysaccharide of gram-negative bacteria, (ii) metabolites of dominant flora such as SCFAs and TMAO, and (iii) intestinal inflammatory factors including TNF-α, IL-1, IL-6, TNF, and iNOS (32, 50, 98, 99, 104). The influx of LPS from gut extravasation into the bloodstream and systemic circulation may be linked to the disruption of the gut mucosal barrier. In addition, studies have shown that the interaction between LPS and TLR induces an inflammatory response in the host, exacerbating damage to gut barrier function (84, 87–90). One study reported that the plasma levels of LPS, D-lactate, zonulin, TNF-α, IFN-γ, and IL-6 progressively increased over time in macaques with IS compared to healthy macaques (50). ICH in mice causes significant intestinal mucosal injury, barrier dysfunction, delay of slight intestinal movement, an inflammatory reaction characterized by white blood cells infiltrating intestinal tissue, and elevated levels of inflammatory cytokines in intestinal tissue and serum.

Furthermore, oxidative stress is distinguished by indirect reactive oxygen species and antioxidant gene levels (32). The cytotoxic effect of inflammatory substances causes intestinal microvilli injury, which results in the breakdown of tight junction structures between cells, a decrease in the tight junction proteins occludin and claudin-1, and an increase in the gut barrier dysfunction (32, 98, 99, 104). After CeVD, dysbiosis of the gut microbiota, intestinal mucosal injury, and chronic systemic inflammation persist and may impair immunological homeostasis.

SCFAs are essential in maintaining gut bacterial balance, gut epithelial functional integrity, gut immunology, and inflammation. They induce the development of the intestinal barrier by suppressing the NOD-like receptor protein 3 (NLRP3) inflammasome and autophagy and protect the intestinal barrier against LPS damage. SCFAs also function as an energy source to protect the intestinal barrier and suppress autophagy and as a histone deacetylase (HDAC) inhibitor to inhibit the NLRP3 inflammasome (105). The interaction between the NLRP3 inflammasome and autophagy affects the intestinal barrier function; however, SCFAs can restore barrier function (105). A reduction in SCFAs following IS has been confirmed. Butyrate is a frequently investigated SCFA metabolite whose supplementation can reduce the poor prognosis associated with IS and improve intestinal barrier dysfunction. In addition to lowering inflammation and damage in the gut, butyrate also affects tight junction proteins (28). ZO-1, occludin, and claudin-4 are upregulated to preserve the integrity of the gut mucosa (46). In addition, butyrate supplementation influences epithelial oxygen consumption and preserves the stability of the hypoxia-inducible factor, a transcription factor that regulates intestinal barrier protection (106).

### Bidirectional interaction of microbial metabolites after CeVD

Altered intestinal permeability, impairment of intestinal barrier function, and the passage of intestinal flora metabolites through the intestinal barrier affect CeVD. The relationship between intestinal bacteria and CeVD may be directly related to the actions of intestinal metabolites, such as TMAO, SCFAs, taurine, and other medications. In patients with IS, plasma TMAO levels were dose-dependently linked to worse functional outcomes (107, 108). TMAO upregulates the expression of scavenger receptors (CD36 and SR-A1) in macrophages. This results in increased cholesterol buildup in macrophages and the production of foam cells in atherosclerotic lesions (109), hence increasing the risk of cerebral thrombosis. In contrast, TMAO can induce excessive vascular inflammation and oxidative stress, reducing the bioavailability of eNOS-derived NO and decreasing endothelial function in elderly rats (108, 110). In contrast, endothelial dysfunction is induced by excess generation of reactive oxygen species, and inflammation is the major mechanism of cerebrovascular injury (108, 110). Moreover, the cascade of mitogen-activated protein kinase activation, extracellular signal-associated kinase, and nuclear factor-B signaling in endothelial cells can induce vascular inflammation (111). Increased proinflammatory intermediate CD14 and CD16 also promote increased serum TMAO concentrations, which enhances the risk of recurrent stroke after IS and cardiovascular events (112). This is done to prevent aseptic inflammation and platelet aggregation in the body and lower the risk of recurrent stroke following stroke (63). Chronic low-dose TMAO influences inflammatory variables, such as TNF and IL-1. However, it reverses LPS-induced BBB damage and memory impairment, prevents the degradation of cell structural arrangement, lowers oxidative stress, and preserves the integrity of cerebrovascular endothelial cells (113). Nonetheless, in ICH, a high blood concentration of TMAO may exacerbate cellular inflammation by activating microglia and astrocytes (114). Furthermore, it does not affect the expression of inflammatory factors around the hematoma in the acute phase of ICH. In addition, it does not affect lesion volume in the acute phase of ICH, brain swelling and brain water content, and later brain atrophy, white matter damage, and nerve function (114). Therefore, TMAO is not a therapeutic target for ICH (114). One of the most significant metabolites of intestinal microbes, SCFAs can affect oxidative stress, intestinal barrier dysfunction, and inflammatory response, and their production of bacterial decrease is associated with IS and ICH (41, 74, 80). The quantity of SCFA-producing bacteria in the gut is susceptible to aging and is related to the severity of IS and poor prognosis conditions such post-stroke depression and cognitive impairment (40, 42, 80, 83). SCFA supplementation can promote post-stroke recovery and cortical reorganization, modulate post-stroke synaptic plasticity, and decrease microglial activation and immune cell production (81). Transplantation of SCFA-producing bacteria can (i) reduce IL-17 T cells in the brain, (ii) alleviate neurological deficits and inflammation following stroke, and (iii) increase the concentration of SCFA in the gut and plasma of elderly stroke mice (37). Moreover, it can increase the expression of the mucin genes Muc2 and Muc4 in the intestinal epithelium. It can also increase the protective mucous layer, modify the T lymphocyte population, modulate antibody secretion, and might penetrate the systemic circulation to regulate cytokine release (37, 115). The transplantation of butyric acid-producing bacteria-rich bacterial flora can minimize nerve damage and cerebral infarction volume following IS (51). Additionally, it can alleviate cerebral edema, lower blood cholesterol levels, inhibit histone deacetylase HDAC, activate PPAR, reduce inflammation, and alter the gut flora (51). This results in an increase in helpful lactic acid bacteria and intestinal barrier repair (51). In conclusion, SCFAs influence CeVD primarily by modulating inflammatory and immunological pathways and intestinal barrier function but not by directly affecting brain tissues. Taurine, a gut microbial metabolite, is closely associated with lesions in IA and IS (35, 116, 117). A rtificial taurine supplementation can reverse the formation and rupture of IA, which is correlated with a decrease in host inflammatory markers, thus weakening the inflammatory process. Reducing the activation of MMPs, particularly MMP-9, inhibits extracellular matrix remodeling, increases collagen IV and laminin in the structure of the cerebral artery, and preserves cerebral artery integrity (35). Moreover, taurine canreverse the activation of PARP and NF-B in the ischemic core penumbra, drastically reducing TNF-α, IL-1β, inducible nitric oxide synthase, and ICAM-1, and decreasing myeloperoxidase activity. Reduced neutrophil infiltration within the penumbra and center (116, 117). Exogenous injection of adequate taurine may restrict intracellular taurine release and increase extracellular taurine levels, thereby promoting intracellular homeostasis, clearing HOCl, reducing Tau-NCl2 synthesis, elevating tau-NHCl levels, and inhibiting inflammation. This eventually leads to a decrease in ischemic brain injury (117). Therefore, taurine reduces the inflammatory response and may protect against secondary injury after CeVD, thus decreasing IA occurrence. Intestinal bacteria metabolize bodily components to produce intestinal metabolites, such as tryptamine, indole, indole propionic acid, indoleacetic acid, indole aldehyde, bile acid, and phenylacetylglutamine. These metabolites can regulate intestinal permeability, gastrointestinal motility, and microbial composition (118–124).

### Other potential mechanisms

In addition to the processes mentioned above, another putative mechanism for cerebrovascular injury is the reciprocal connection between gut microorganisms and the brain. The intestine can influence the brain in various ways, including the neuroendocrine pathway, anatomical pathways, neurotransmitters, and many other mechanisms (125, 126). Anatomically, the vagus nerve in the spinal cord and autonomic nervous system allows the intestine to communicate directly with the brain. The enteric neural system in the stomach and autonomic nervous system, and the vagus nerve in the spinal cord can both contribute to bidirectional communication between the gut and brain (125, 127–129). Accordingly, gut microorganisms can directly stimulate afferent neurons of the enteric nervous system and vagal signals of the gut to trigger inflammatory reactions and affect the brain (130). The neuroendocrine-hypothalamic-pituitary-adrenal axis is a pathway by which gut microorganisms influence the brain (125, 129). Gut microbes also play crucial neuroendocrine roles. Changes in the dominant microbiota affect the expression and release of the hypothalamic adrenocorticotropin-releasing hormone, which affects adrenal gland function (125, 129). *Bifidobacteria* in feces can influence the stress response in GF mice (131).

Additionally, being under a lot of stress causes the amount of *Bacillus mimicus* to drop and the amount of *Clostridium perfringens* to increase (132). The brain is also affected by neurotransmitters, neuromodulators, and other chemicals produced by gut microbes (125, 133, 134). For instance, chemicals produced by gut microorganisms and intestinal bacteria, such as gamma amino acids, butyric acid, 5-hydroxytryptamine (5-HT), dopamine, and SCFAs, can ultimately affect the brain through numerous exchanges and interactions (135). For example, the fish gut bacterium *Edwardsiella tarda* consumes tryptophan from food and breaks it down to produce indole, which activates Trpa1 channels in enteroendocrine cells and causes the production and secretion of 5-HT (136, 137). Likewise, serotonin stimulates intestinal neurons, improves intestinal dynamics, and stimulates vagal activity, all of which affect brain activity. Additionally, gut microbes control how tryptophan precursors produced by 5-HT are synthesized, influencing how neurons that release pentraxins in the brain communicate and, ultimately, how neurological and psychiatric illnesses develop (138, 139). To ascertain whether these possible pathways are pathophysiological mechanisms of cerebrovascular injury caused by gut bacteria, further in-depth research into each pathway may be worthwhile.

## Intervention model, efficacy, and application prospect of the gut-brain axis

### Dietary intervention

**Figure 5:**
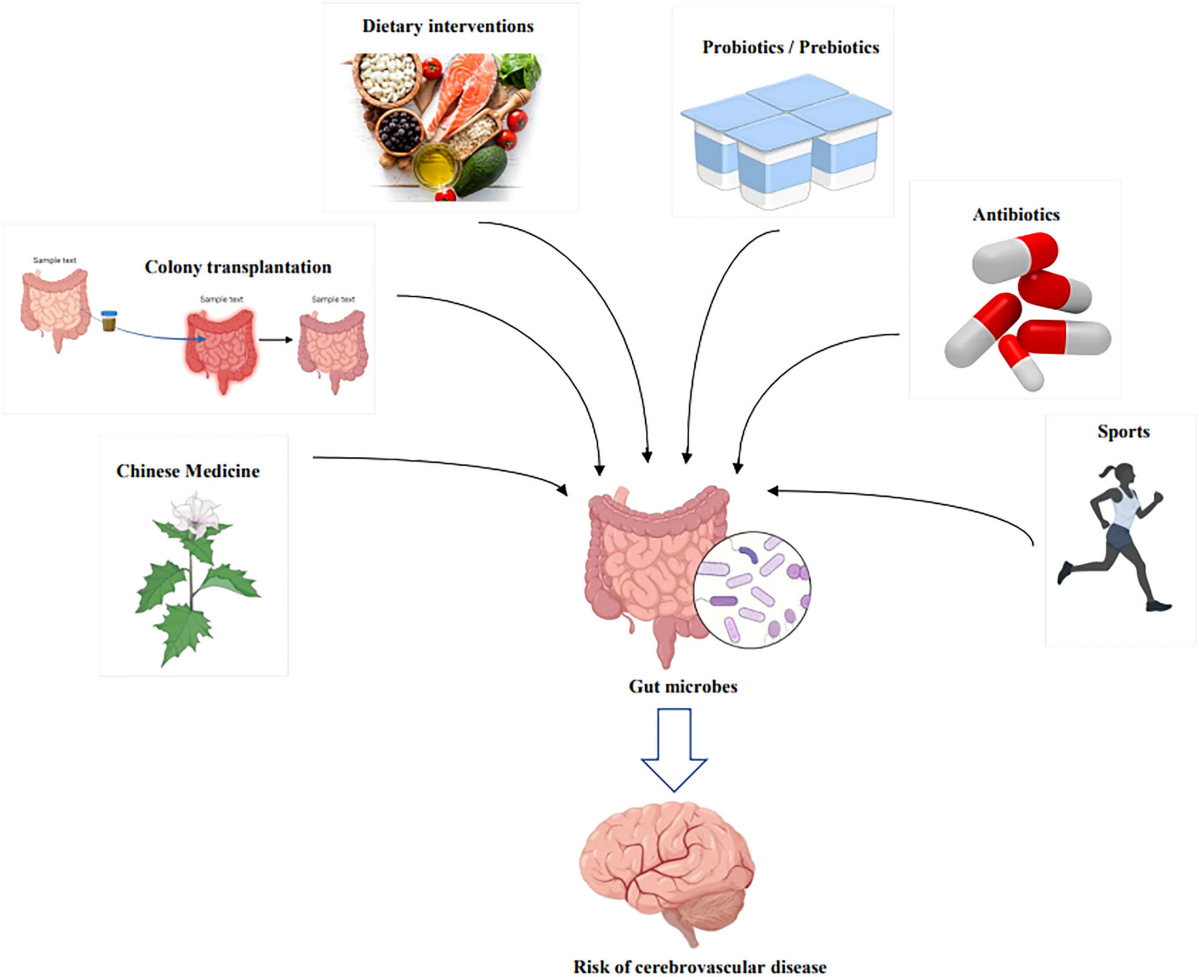
Therapeutic modalities for modulating the risk of cerebrovascular disease through intervention with gut microbes. By Figdraw and BioRender.com.

Diet is one of the direct drivers of gut microbial dysbiosis, affecting the course of cardiovascular disease and CeVD, as indicated in **Table 1** and **Figure 5**. In addition, numerous scientific studies have indicated the importance of diet and nutrition in reducing cardiovascular diseases and CeVD (140, 146). Diets rich in fat and carnitine, such as meat and eggs, increase the abundance of TMAO-producing gut bacteria, which are believed to promote TMAO-induced platelet activation and increase the risk of thrombotic events (147). Furthermore, dietary choline can boost the activation of microglia and astrocytes in the vicinity of the hematoma in ICH, causing inflammation (114); when TMAO levels in the blood surpass a quarter of the average, the risk of stroke and cardiovascular disease increases by 2.5 times (148, 149). In addition, the dominant flora following an intestinal microbiome disorder metabolizes amino acids and produces harmful metabolites such as para-cresol sulfate, horse uric acid, indolyl sulfate, para-cresol glucosidic acid, phenylacetylglutamine, and phenyl sulfate. These metabolites cause secondary renal damage when excreted by the kidney (18, 150). Therefore, those at risk of stroke should minimize meat and egg yolk consumption (18).

**Table 1:** Interventions, influence mechanisms and efficacy of gut microbes in cerebrovascular disease.

	Interventions	Mechanism	Study object	Curative effect
Dietary interventions	Choline diet	1. Increase TMAO to increase platelet activation and thrombotic events.	1. C57 mice (114)	Increase risk of IS and ICH
			2. Human (45) (112)	
		2. Increase the number of microglia, astrocytes, and inflammatory mediators CD14 and CD16.	3. Human + Germ-free mice (126)	
	Ketogenic diet	1. Regulate intestinal flora in epileptic and multiple sclerosis mice.	1. Wistar rat	Improve the prognosis of stroke, but whether it is affected by gut microbes needs further study
		2. Alleviate excitatory toxicity, oxidative stress, and apoptotic events.	(132)	
			2. C57 mice	
			(130)	
			3. Human	
			(131)	
	High sugar diet	1. Reduce the ratio of Firmicutes to Bacteroidetes in intestinal microorganisms.	1. C57 mice	Reduce the risk of IS.
		2. Increase medium and small particle sizes of high-density lipoprotein (HDL) cholesterol.	(135)	
	High-fiber diet	Increase blood levels of short-chain fatty acids and decreases the inflammatory response.	1. Human (138)	Reduce the risk of IS.
Fecal bacteria transplantation	Fecal bacteria transplantation	1. Aid in restoring the equilibrium of the gut microbiome.	1. C57 mice	

	
	
				Reduce the risk of IS, ICH, IA
		2. Reduce the negative effects of inflammation, increase intestinal T cells that are protective, and decrease Treg cells and IL-17-producing T cells.	(37) (99)	
	
		3. Restore occludin and claudin-1 to improve the function of the intestinal barrier.	2. Human (29) (63)	
		4. Keep the extracellular matrix of cerebral vascular arteries in good shape.	3. SD rat (140)	
	Prebiotics, probiotics	1. Aid in re-establishing the balance of the gut microbiota.	1. Human (28)	Improve anxiety, depression, and other emotional problems after stroke
		2. Decrease intestinal TMAO synthesis and increased short-chain fatty acid production.		
		3. Minimize intestinal barrier and tight junction protein damage.		
		4. After a stroke, lessen the harm caused by inflammation and the activation of the innate and adaptive immune systems.		
Traditional Chinese Medicine	Tanhuo decoction	1. Increase the complexity of intestinal microbiome symbiosis and competition between LPS-producing bacteria and opportunistic pathogenic bacteria.	1. Human (63)	Reduce the risk of IS.
		2. LPS biosynthesis, TMA, aseptic inflammation, and platelet aggregation were reduced in IS.		
	Rhubarb anthraquinone glycosides	1. Control the intestinal microbial imbalance brought on by IS and prevent the imbalance brought on by cerebral ischemia.	1. SD rat (127) (141)	Ameliorate the injury of cerebral ischemia-reperfusion
		2. The levels of aspartic acid and glutamate content were decreased and 5-hydroxytryptamine, 5-hydroxy indole acetic acid, and -aminobutyric acid were elevated in the brain and colon, balancing the imbalance between neurotransmitter release and breakdown brought on by intestinal flora alteration.		
	Tongqiao Huo Xue Soup	Change the main components of intestinal flora in stroke rats, reduce the excessive increase of Bacteroides, regulate the abnormal abundance of some bacteria, regulate T cell imbalance, inhibit the inflammatory response, and improve intestinal barrier injury.	1. SD rat (36)	Ameliorate the intestinal microbiota disorder and induced inflammatory response after IS
	Decoction of Pueraria root and cymbidium root nodules combination	1. Control the dysbiosis of the intestinal microbiome and raise the levels of ZO-1 and claudin-5 to safeguard the gut barrier.	1. SD rat (142)	Alleviate the damage of IS
		2. By lowering the concentrations of D-lactic acid, lipopolysaccharide, and diamine oxidase to lessen intestinal flora translocation and harm to gut barrier function.		
		3. Reverse dyslipidemia lowers thrombosis risk and blood viscosity.		
	NaoMaiTong	Regulate intestinal microorganisms and microbial metabolites, inhibiting Enterobacteriaceae to repair the intestinal barrier and promote the prognosis of stroke	1. SD rat (44)	Improve the prognosis of IS
	Xinglou Chengqi Decoction	The regulation of short-chain fatty acid (SCFA) production bacteria, such as verrucous colitis and Akkermansia, as well as the regulation of inflammation-causing bacteria, such as para-vortreponema, Roseburia, Streptophyta, and Enterococcus, leads to an increase in SCFA levels and the release of anti-inflammatory compounds like IL-10. Additionally, it reduced the expression of pro-inflammatory molecules such as TNF-α, IL-17A, and IL-22.	C57 mice (143)	Improve nerve function, alleviates cerebral infarction, and reduces neuronal apoptosis
		Increase the production of vasoactive intestinal peptide and its receptor to increase BBB permeability and lessen the degradation of VE-calcic protein, Claudin-5, ZO-1, and occludin. inhibits MMP 2/9 expression in cerebral microvascular endothelial cells to improve endothelial junction complex and encourage transendothelial resistance.	1. SD rat (144)	Improve BBB integrity after IS
	Resveratrol	Reduce systemic inflammation and neuroinflammation after stroke by modifying intestinal microbiome-mediated alterations in Th17/Tregs and Th1/Th2 polarity in the small intestine.	C57 mice (31)	Nerve damage after protecting IS
Antibiotics	Broad-spectrum antibiotic	1. Regulation of inflammation and reduction of IL-1, IL-6, iNOS, and other inflammatory factors in the gut.	1. C57 mice	Reduce the formation of intracranial aneurysms.
		2. Immune homeostasis in the small intestine is changed by changing dendritic cell activity, leading to an increase in regulatory T cells and a decrease in IL-17-producing T cells.	(93) (87) (34)	Reduce inflammation after IS but using antibiotics as a long-term treatment method may increase the risk of IS and intracranial aneurysms.
			2. SD rat (145)	
Other	Exercise training	Inhibit the production of IL-2 and IL-6, encourage the conversion of microglia into M2, control intestinal flora, and lessen the likelihood of post-stroke depression.	SD rat (91) (71)	Reduce the incidence of post-stroke depression.
		Ionize calcium-binding adaptor molecule 1, tumor necrosis factor-alpha, monoclonal MHC Class II RT1B, and dramatically reduce other inflammatory markers and the inflammatory microbiota BAC303, EREC482, and LAB158.		Improve the prognosis of IS.
	Ghrelin	Inhibit ICAM-1 expression and endotoxin translocation, reduce intestinal mucosal injury and decrease intestinal permeability.	C57 mice (102)	Reduce intestinal barrier dysfunction after ICH.

IS, ischemic stroke; ICH, intracerebral hemorrhage; IA, intracranial aneurysm; TMAO, trimethylamine N-oxide; LPS, lipopolysaccharide; TAM, trimethylamine.

In antiepileptic mice, a ketogenic diet was observed to decrease gut bacterial diversity and increase the relative abundance of *Akkermansia muciniphila* (151). In addition, the short-term gut microbiota of mice with multiple sclerosis is dysregulated, and a long-term ketogenic diet leads to a progressive decrease in *Akkermansia* bacteria (152). The amount and variety of gut flora were restored after 12–24 weeks of a ketogenic diet (152). By reducing excitotoxicity, oxidative stress, and apoptotic processes, a ketogenic diet can improve post-stroke motor function and increase the neuronal repair of the ischemic penumbra in patients with IS (142, 143). Further research is necessary to determine whether a ketogenic diet can prevent and treat CeVD by modulating gut bacteria.

In addition, one study reported that a diet high in sugar calories significantly impacted CeVD. In the barley diet group, the ratio of *Bacteroidetes* to *Firmicutes* in intestinal microorganisms was significantly lower than that in the rice diet group. In contrast, the cholesterol level of medium and small particle sizes of high-density lipoprotein (HDL) was significantly increased, which may decrease the risk of stroke (144). Intestinal microbiota abnormalities also result in the loss of SCFAs, whereas a high-fiber diet can increase butyrate levels in the circulation (145). In mice fed a high-fiber diet, inflammation generated by endotoxins and lipopolysaccharides increased IL-1RA, whereas IL-1 and tumor necrosis factor-alpha (TNF-α) were reduced. By polarizing Th2 in mice and increasing the substitution activation of macrophages, endotoxin- and lipopolysaccharide-induced illness damage is reduced, which helps enhance brain immunity (141, 153). The incidence and risk of stroke are negatively correlated with dietary fiber intake (154).

### Fecal bacteria transplantation

Flora transplantation restores the original dominant intestinal flora and intestinal homeostasis by transferring functional bacteria from the normal population feces to the gastrointestinal tract of dysbiosis, thus achieving the objective of treating the pathological state in the host (155). Currently, CeVD animal models have been used to investigate the therapeutic effects of this method (29, 35, 37, 46, 63, 68). Intestinal T cells develop protective activity following transplantation of feces from a young population into mice with IS. Treg cells and IL-17 T cells contribute to decreased inflammation, neurological deficits, and impairment of intestinal barrier function following stroke (29, 37, 63). Transplantation of adult rat bacterial flora into reproductively aged rats significantly improved the area of cerebral infarction, lowered the production of IL-17, and exacerbated behavioral impairment after stroke (68). *H. hathewayi* is positively associated with taurine levels, decreases cerebral artery inflammation, and restores extracellular matrix integrity to avoid IA (35). In mice with ICH, transplantation of bacterial flora can affect T cells in the brain, reduce neuroinflammation following bleeding, and restore the average fluorescence intensity of the tight junction proteins occludin and claudin-1, thereby restoring intestinal barrier function (46). Flora transplantation plays an effective role in the prevention and treatment of CeVD in animal models. However, there are difficulties in implementing simple flora transplantation applied clinically to feces collected from healthy donors or the recipients themselves (self-FMT) and then administered to the intestines of patients with the disease or associated ecological disorders (124).

Therefore, it is of major application value to test medications with comparable efficacy or generate treatment-specific microbiota. Metformin possesses potent anti-inflammatory and immunosuppressive properties. In ICH mice, metformin effectively ameliorates neurological dysfunction and lowers neuroinflammation by suppressing the proinflammatory polarization of microglia/macrophages and has a neuroprotective effect similar to that of microflora transplantation (48). The most widely studied florae are prebiotics and probiotics. Probiotics are non-digestible compounds that regulate the composition and/or activity of the intestinal microbiota through their metabolism by microorganisms in the gut, thereby conferring beneficial physiological effects to the host (156).

In contrast, probiotics are live, giveable microorganisms capable of conferring health benefits to the host (157). After intake, probiotics and prebiotics can be transported straight to the gastrointestinal system, inhibiting the action of pathogenic gut microorganisms and restoring the balance of “beneficial bacteria,” “neutral bacteria,” and “bad bacteria” to prevent and treat disease (158, 159). For example, probiotics administration restored the intestinal microecological balance, intestinal TMAO production decreased, SCFA production increased, tight junction protein damage decreased, and adaptive and innate immune activation decreased. In addition, inflammatory damage following stroke decreased (82, 160, 161). In addition, it has been proven to treat mood disorders following a stroke, including anxiety and depression, 3 months after the event (28).

### Traditional Chinese medicine

Chinese medicine is also an important intervention for gut microbes and CeVD. Tanhuo decoction can control gut microorganisms, enhance gut bacteria, compete with harmful bacteria to decrease LPS and TMAO synthesis, and decrease aseptic inflammation and platelet aggregation in blood vessels and the neurological system (63). Anthraquinone glycoside rhubarb is an efficient treatment for cerebral ischemic injury and can regulate the disturbance of gut flora induced by cerebral ischemia-reperfusion injury (82). Consequently, there is an imbalance between the release and degradation of neurotransmitters such as 5-HT and 5-hydroxyindoleacetic acid, which is caused by disturbances in the intestinal flora (82). The classic TCM formula Tongqiao Huoxue Decoction (TQHXD) can (i) control the changes in flora after IS, (ii) reduce the excessive increase in *Bacteroides*, (iii) control the abnormal changes in the abundance of some flora, (iv) improve the inflammatory response caused by T cell imbalance, and (v) restore the function of the gut barrier (18). In addition, decoctions, including Pueraria root and rhizome, ameliorated dyslipidemia, elevated blood viscosity, and a high risk of thrombosis after stroke and repaired the ecological imbalance of the gut microbiome and the integrity of the brain-gut barrier (162). NaoMaiTong corrected the dismal prognosis following stroke by restoring gut flora (44). Formulations of Xingu Chengqi stimulate the release of anti-inflammatory factors, such as IL-10, and downregulate proinflammatory factors, such as TNF-α, IL-17A, and IL-22 (163). Increasing SCFA levels also enhance neurological function, alleviates cerebral infarction, and reduce neuronal death (163). Resveratrol mitigates ischemic brain injury by modulating T lymphocytes to minimize BBB injury and neuroinflammation caused by proinflammatory factors (31). Shengli Sansheng Pulvis improves the endothelial connection complex by increasing vasoactive intestinal peptides and receptors and decreasing claudin-5, ZO-1, occludin, VE-calcitonin, and MMP 2/9 expression. Moreover, it increases trans-endothelial resistance in microvascular endothelial cells of the brain following ischemia (164). In conclusion, modulation of intestinal bacteria by TCM has a substantial influence on the treatment of CeVD and has a substantial therapeutic effect.

### Antibiotic therapy

Antibiotic intervention can alter the makeup and function of the intestinal microbiota and microbiome homeostasis. The use of antibiotics to consume gut flora, which produces inflammatory factors such as IL-1, IL-6, and iNOS, decreases IA formation (93). In IS, gut microbial changes generated by antibiotic intervention can induce dendritic cell modifications that affect gut immune homeostasis, resulting in increased regulatory T cells, decreased IL-17 T cells, and reduced ischemic brain injury in mice (87). In contrast, depletion of gut microbes by broad-spectrum antibiotics does not affect the area of brain damage 1 day after stroke but suppresses systemic immunity and reduces survival from IS and severe secondary colitis (34). In addition, broad-spectrum antibiotic treatment for 3 weeks reduces eNOS activity, leading to (i) brain endothelial cell dysfunction, (ii) a significant increase in spontaneous tone in the middle cerebral arteries (MCAs), (iii) significant blunting of L-NAME-induced vasoconstriction, (iv) reduction in intraluminal ATP-mediated dilation, and (v) significantly increased risk of stroke and IA (165). Therefore, antibiotic administration to prevent and cure strokes caused by gut bacteria remains controversial. However, applying antibiotics with a limited spectrum that target specific bacteria may result in more precise CeVD prevention and treatment.

### Other

Exercise can increase learning, enhance memory, and alleviate despair, anxiety, and other negative feelings. In addition, exercise training can block the proinflammatory factors IL-2 and IL-6, accelerate the transformation of microglia into M2, regulate gut flora, and lower the incidence of depression following stroke (91). It can also increase the decline in *BAC303*, *EREC482*, and *LAB158*, which are inflammatory microbiota. This alleviates behavioral impairment after stroke, reduces infarct size, and improves infarct cell survival rate (166). In addition, ghrelin reduces ICH-induced intestinal barrier dysfunction by reducing intestinal mucosal damage, lowering intestinal permeability, enhancing tight-junction molecules, inhibiting ICAM-1 expression, and suppressing endotoxin translocation (102).

## Limitations and prospects

There are still many unanswered questions regarding the gut microbiota and pathogenesis of CeVD. First, the ultimate goal of scientific research is to cure clinical disorders. However, the prevention and therapy strategies for intestinal bacteria in CeVD are not specific, and targeted flora transplantation has not been effectively utilized in medical practice. In the future, evaluation of the efficiency of interventions against specific pathogenic bacteria may be of greater clinical relevance. Second, numerous studies have demonstrated an association between gut microbial diseases and CeVD. However, further research is needed to confirm the role of gut microbes in the occurrence of CeVD. Prevention and therapy can be enhanced if this causal relationship can be confirmed early. In addition, the primary mechanisms of action of intestinal microorganisms in CeVD include immunological problems, inflammatory reactions, gut barrier dysfunction, and the impact of gut metabolites. However, the specific molecules, locations, and mechanisms of gut bacteria acting on CeVD after imbalance have not been explored. If these constraints can be overcome, the mechanism and potential treatment of intestinal bacteria that cause CeVD can be developed, and the therapeutic significance of this research can be significantly enhanced.

## Conclusion

Gut bacteria provide novel ideas for the effective prevention and treatment of CeVD, thus reversing the conventional understanding of vascular diseases and neuroinflammation. In the link between gut bacteria and CeVD, metabolites of dysbiosis of the gut flora, such as TMAO, SCFAs, and taurine, contribute significantly to cerebrovascular injury and increase disease progression. The food consumed may affect these metabolites; hence, dietary changes may be related to an increased risk of CeVD. In managing gut microorganisms and CeVD, dietary treatments, FMT, herbal medication, and antibiotics have specific protective effects on the regulation of gut microbes and the prevention of CeVD. However, research on the association between gut microorganisms and ICH, IA, CSVD, and CCM is still in its infancy. Future studies examining gut-brain axis-based CeVD risk reduction and therapeutic targets are of great clinical significance.

## Data availability statement

The original contributions presented in the study are included in the article/**Supplementary Material**. Further inquiries can be directed to the corresponding authors.

## Author contributions

LZ and XZ designed the research and determined the structure of the manuscript. XZ, LX, LW selected the references and contributed to the writing. LZ and SW contributed to the revision and finalization of the review. All authors contributed to the article and approved the submitted version.

## Funding

This study was supported by the National Science & Technology Fundamental Resources Investigation Program of China to LZ (No.2018FY100900), The Hunan Provincial Natural Science Foundation of China Grant to YZ (No.2021JJ30923), The Provincial Science and Technology Innovation Leading Talents Project to LZ (No.2021RC4014), National Clinical Research Center for Geriatric Disorders (XiangYa Hospital).

## Acknowledgments

We thank all research on gut microbes and cerebrovascular diseases included in this study.

## Conflict of interest

The authors declare that the research was conducted in the absence of any commercial or financial relationships that could be construed as a potential conflict of interest.

## Publisher’s note

All claims expressed in this article are solely those of the authors and do not necessarily represent those of their affiliated organizations, or those of the publisher, the editors and the reviewers. Any product that may be evaluated in this article, or claim that may be made by its manufacturer, is not guaranteed or endorsed by the publisher.
