# Supplementary material for: Gut microbes in cerebrovascular diseases: Gut flora imbalance, potential impact mechanisms and promising treatment strategies
Source: Front Immunol. 2022 Oct 31;13:975921. doi: 10.3389/fimmu.2022.975921 (PMC9659965; doi:10.3389/fimmu.2022.975921)
Supplement: Supplementary file 1 [file Table_1.docx]

| **Bacteria** | IS | ICH | IA | CSVD | CCM |
| --- | --- | --- | --- | --- | --- |
| **Actinobacteria** |  |  |  |  |  |
| Bifidobacterium | Human↑ [163] [164][38][40], Human↓ [67] |  |  |  | Human ↓ [71] |
| Actinobacteria | Rat↓ [32] | Human↑ [47] |  |  |  |
| Atopobium | Human↑ [165] |  |  |  |  |
| Actinomyces | Rat↑ [166] |  |  |  |  |
| Coriobacteriales |  | Rat↓ [47] |  |  |  |
| **Bacteroidetes** |  | Rat ↓ [69] |  |  |  |
| Prevotella/ Prevotellaceae | Monkey↑ [90] Human↑ [167][168] Rat↑ [142]  Rat↓ [159] Human↓ [35][164] |  |  |  |  |
| Bacteroidales S24-7 | Human↓ [66] |  |  |  | Rat↑ [90] |
| Odoribacter | Human↑ [58] |  |  |  | Human ↑ [71] |
| Roseburia | Human↑ [65][167], Human↓ [38] [48] |  |  |  |  |
| Bacteroides | Human↑ [167] Rat↑ [104][57] Human↓ [35][164][48] |  |  |  |  |
| Porphyromonadaceae | Human↑ [48] |  |  |  |  |
| Flavobacteriaceae | Human↑ [58] |  |  |  |  |
| Alistipes | Rat↑ [104][57] |  |  |  |  |
| Parabacteroides | Human↓ [164] Human↑ [167][40] Rat ↑ [57] |  |  |  |  |
| Bacteroidetes | Human↓ [40] Rat↓ [32] | Rat↓ [69] |  |  |  |
| **Firmicutes** | Rat↓ [170][32] | Rat↑ [69] |  |  |  |
| Lactobacillus | Human↑ [40][38] Monkey↓ [90] Rat↑ [166] |  |  |  |  |
| Faecalibacterium | Human↑ [58] Rat↑ [104]  Human↓ [35][48][164][65] Monkey↓ [90] |  |  |  | Human ↓ [71] |
| Hungatella hathewayi |  |  | Human ↓ [66] |  |  |
| Peptostreptococcaceae | Rat↑ [166] | Rat ↑ [33] |  |  |  |
| Romboutsia | Rat↑ [59][57] | Rat↑ [33] |  |  |  |
| Enterococcus | Human↑ [67][40][37] Rat↑ [66] | Human↑ [37] |  |  |  |
| Lachnospiraceae | Rat↑ [162] [66] Human↓ [38] [48] |  |  |  |  |
| Erysipelotrichaceae | Human↑ [65] Rat↑ [66] |  |  |  |  |
| Blautia | Human↑ [163] Human↓ [48] |  |  |  |  |
| Anaerostipes | Human↓ [48] |  |  |  |  |
| Lactobacillaceae | Human↓ [48] |  |  |  |  |
| Ruminococcaceae | Human↑ [58] Human↓ [38] Rat↓ [66] |  |  |  |  |
| Christensenellaceae | Rat↑ [66] |  |  |  |  |
| Peptoniphilus | Human↑ [37] | Human↑ [37] |  |  |  |
| Ezakiella | Human↑ [37] | Human↑ [37] |  |  |  |
| indolis | Rat ↑ [20] |  |  |  |  |
| neopropionicum | Rat ↑ [20] |  |  |  |  |
| cellulolyticum | Rat ↑ [20] |  |  |  |  |
| cellulovorans | Rat ↑ [20] |  |  |  |  |
| bolteae | Rat ↑ [20] |  |  |  |  |
| populeti | Rat ↑ [20] |  |  |  |  |
| Megasphaera | Human↑ [35][38] |  |  |  |  |
| Oscillibacter | Human↑ [35] |  |  |  |  |
| Ruminococcus | Rat↑ [170][166] Human↑ [164][167] |  |  |  |  |
| Megamonas | Human↑ [164][167][163] |  |  |  |  |
| Holdemanella | Human↑ [163] |  |  |  |  |
| Clostridium | Human↑ [163] Rat↑ [166] |  |  |  |  |
| Papillibacter | Rat↑ [104] |  |  |  |  |
| Peptococcaceae | Rat↑ [169] |  |  |  |  |
| Oscillospira | Monkey↓ [90] Human↓ [38] |  |  |  |  |
| Dialister | Human↑ [164][167] |  |  |  |  |
| Lactobacillus ruminis | Human↑ [165] |  |  |  |  |
| Lactobacillus sakei | Human↓ [165] |  |  |  |  |
| Veillonella | Human↑ [38] |  |  |  |  |
| Acidaminococcus | Human↑ [38] Rat↑ [38] |  |  |  |  |
| Lachnospira | Human↓ [38] |  |  |  |  |
| Streptococcus infantis | Human↑ [168] |  |  |  |  |
| Mogibacteriaceae | Rat↑ [66] |  |  |  |  |
| Eubacterium | Human↑ [40] |  |  |  |  |
| RF39 |  | Human [47] |  |  |  |
| TANB77 |  |  |  |  |  |
| Anaerotruncus | Rat↑ [57] |  |  |  |  |
| **Proteobacteria** | Rat↑ [170] Human↑ [38] |  |  |  |  |
| Campylobacter |  |  | Human [171  ] ↑ |  |  |
| Enterobacteriales | Human↑ [63] [48] [65] Rat↑ [66] | Rat ↑ [33] |  |  |  |
| Escherchia | Rat↑ [59][166] Human↑ [67] | Rat ↑ [33] |  |  |  |
| Shigella | Rat↑ [59][166] |  |  |  |  |
| Gammaproteobacteria | Human↑ [63] |  |  |  |  |
| Escherichia/Shigella | Human↑ [37] | Human↑ [37] |  |  |  |
| Desulfovibrio | Human↑ [35][40] |  |  |  |  |
| Klebsiella | Rat↑ [104] Human↑ [40] |  |  |  |  |
| Shuttleworthia | Rat↑ [104] |  |  |  |  |
| Haemophilus | Rat↑ [104] |  |  |  |  |
| Proteus | Rat↑ [104] |  |  |  |  |
| Sutterella | Human↑ [38] |  |  |  |  |
| Escherichia | Human↑ [167] [164] |  |  |  |  |
| Brevundimonas | Rat↑ [166] |  |  |  |  |
| **Verrucomicrobia** | Human↑ [35] |  |  |  |  |
| Akkermansia | Human↓ [164], Rat↑ [59][20][57], Human↑ [58] [48] | Human↑ [47] |  |  |  |
| **Deferribacteres** |  |  |  |  |  |
| Deferribacteres | Rat↑ [170] |  |  |  |  |
| **000Lentisphaerae** |  |  |  |  |  |
| Victivallis | Human↑ [58] |  |  |  |  |
| **Candidatus Parcubacteria** |  |  |  |  |  |
| Parcubacteria | Human↑ [58] |  |  |  |  |
| **Patescibacteria** |  | Rat↑ [69] |  |  |  |
| **Tenericutes** |  | Rat↑ [69] |  |  |  |
| **Fusobacteria** |  | Rat↓ [69] |  |  |  |
| **Acidobacteria** |  | Rat↓ [69] |  |  |  |
| **Verrucomicrobia** |  | Rat↓ [69] |  |  |  |
| Firmicutes/Bacteroidetes | Rat↑ [50][47][49][48] |  |  |  |  |
